# Analytical approach to piezoelectric model synthesis with the use of Cauer’s method for system design

**DOI:** 10.12688/f1000research.140943.1

**Published:** 2023-10-05

**Authors:** Jacek Harazin, Andrzej Wróbel

**Affiliations:** 1 Faculty of Mechanical Engineering, Politechnika Slaska, Gliwice, Silesian Voivodeship, 44-100, Poland

**Keywords:** piezoelectricity, mathematical model, synthesis

## Abstract

Background: Piezoceramic materials have unique property which enables direct and bilateral conversion between mechanical and electrical energy. This ability facilitates significant miniaturisation of technology and opens many opportunities in design of new actuators and energy harvesters. Mathematical modelling of piezoelectric modules is notoriously hard due to complex constitutive equations defining mechanical and electrical energy conversion.

Methods: The article presents research on a new synthesis method based on the Cauer’s method for electrical and mechanical system design. Mechanical damping is introduced with the use of Rayleigh’s approximation. A discrete electromechanical model is formed based on the Mason’s piezoelectric model. As an additional element of the research, a non-standard model analysis method using edge graphs and structural numbers is employed to investigate potential reductions in computational requirements for model analysis.

Results: The electromechanical model analysis has shown an error of 5% in relation to the resonance target. The calculated peak in magnitude of displacement at the resonant frequency was well above the capability of a 3.5mm thick piezoelectric plate. The proposed non-standard method of analysis gave identical results in terms of obtained resonance frequencies. The calculated magnitude of vibrations was varying by 50 – 58%.

Conclusions: The synthesis method presented in this paper allows an approximation of piezoelectric parameters of a real system based on the created mathematical model. Currently this method is subject to a considerable error in the determined vibration amplitudes of the system, but it allows a coarse approximation of the parameters while having a very limited number of input data. The additional method of analysis based on structural numbers offers a promising alternative to matrix calculations but requires a more thorough investigation of the computational power required to determine whether it can compete with existing algorithms.

## Introduction

One of the purposes of mathematical modelling in case of physics is to give a clear and systemic picture of relations and energy flow between elements of a system. Physicists use mathematical modelling to describe phenomena and problem solve the mechanics of a wide variety of systems. ^1^ Mathematical synthesis supported by previous empirical analysis of underlying phenomena helps in creating models which describe real behaviour of materials and components with mathematical correlations. Deep understanding gained by thorough analysis can be later used in development of highly sophisticated systems that are used for precise control of various system variables. ^2^ Systems that precisely control room temperature or humidity, systems responsible for engine control and its smooth acceleration or deceleration or systems used for active vibration cancellation are only few of the examples. ^3^^–^^6^ Polynomial equations are great mathematical tools for approximation and interpolation of numerous phenomena which is why they are commonly used in mathematical modelling. ^7^^–^^9^^,^^10^ Laplacian and Lagrangian algebra rely on polynomial and exponential equations to systemically represent components of force and energy having influence on successive derivatives of displacement or charge. Polynomial equations are among the main set of tools in control theory ^2^^,^^7^^–^^9^^,^^11^^–^^16^ and were used during the development of signal filters composed of RLC (R – resistor, L – coil, C – capacitor) components. ^17^^,^^18^

Wilhelm Cauer was among the pioneers of electrical model synthesis, creating algorithms based on transfer function polynomials, defining large range of electrical systems by using mathematical decomposition. Cauer synthesis ^17^^,^^18^ relies on decomposing transfer function polynomials into continued fractions. The set of coefficients of a continued fraction represents values defining each system component properties (like impedance or capacitance). This procedure allows any transfer function of a complex system to be broken down into a series of components represented by a lumped model. Moreover, because of existing analogies between electrical and mechanical elements, this method can be applied to both electrical and mechanical models. ^7^^–^^9^^,^^11^ Piezoelectric materials exhibit properties that can be described with a combination of interactions between both electrical and mechanical components. ^19^ A set of constitutive equations for piezoelectricity ^12^ has been established to describe electromechanical coupling for simple and inverse piezoelectric effect. Because of the complex set of dimensional relations, described by tensors, mathematical modelling of piezoelectric components is being focused mainly on the analytical aspect of this phenomenon. Researchers largely focus on modelling simple piezoelectric components with lumped models ^13^^,^^14^ and matrix tensor equations ^20^ or try to approach the problem using FEM (finite element method) analysis. ^21^ There are also analytical methods that can be labelled as “non-classical” which rely on AI (artificial intelligence) algorithms or graphical representations of the system like “edge graphs”. Many of the developed models are used on a case-by-case basis where system variables defining piezoelectric behaviour are fully or partially known or they are being empirically determined. ^15^^,^^21^^–^^27^ There is a small research gap when it comes to the subject of a full piezoelectric system synthesis where majority of the initial parameters are unknown. This article tries to focus on the development of a new method for piezoelectric system synthesis where the only initial information comes in the form of established resonant and anti-resonant frequencies. The algorithm based on Cauer synthesis method is used to construct an initial discrete mechanical model which serves as the base for piezoelectric model synthesis. Mechanical damping is introduced to the model with the help of Rayleigh’s method. ^28^ The transition between purely mechanical model and a discrete electromechanical model of the piezoelectric plate is done with the combination of Mason’s model and analogies between mechanical and electrical components. There are many unknown piezoelectric parameters which have to be determined in this process. To solve this problem, authors have chosen an approach where most of the parameters are being approximated by using the obtained mechanical properties of an already synthesized system and a set of equations derived from the constitutive equations of piezoelectricity.

Part of this article has been devoted to a study on the potential reductions in computational requirements for model analysis that might be possible when using edge graphs. ^7^^,^^8^^,^^11^^,^^29^ Some methods based on edge graphs bare close naming resemblance like the ones used in ^15^^,^^22^ which may cause confusion. As a matter of clarification, this article is focusing on the method using edge graphs in combination with structural number algebra. Structural numbers visually resemble matrix calculation as each structural number construct consist of rows and columns of numbers. The number of rows and columns, however, is tightly connected to the shape of an edge graph which a given structural number relates to. Each node in an edge graph and all its connections are represented by a single structural number vector ( *e.g.*,|1 2 3 4|for a node connecting four edges numbered from 1 to 4). A structural number is then created by multiplying such node vectors by themselves according to structural number algebra. Arithmetic and logic operations based on structural number algebra were described in depth by professor Stanisław Bellert in his book. ^29^ More details can also be found in previous article published by authors. ^30^ The article also directly relates to adaptations of this method used by other researchers for analysis and synthesis of various systems. ^2^^,^^11^^,^^16^

## Methods

### Mechanical model synthesis

The synthesis process begins with a selection of predetermined resonant and anti-resonant frequencies. The set of frequencies may be freely defined according to future applications or may result from a vibration spectral analysis of the object of interest. In this method, the identified set of frequencies is the only input information. It is used to generate a transfer function polynomial, which can later be transformed into a chain fraction equation. Cauer’s algorithm mentioned in Refs. 8 and 11 was used to create four different types of mechanical models depending on the allocation of coefficients in the chosen transfer function polynomial. Depending on the polynomial, the cascade model resulting from the chain fractioning process can be constrained on one end or not constrained at all. Additionally, the model can be excited by either a kinetic or dynamic force. A combination of those factors creates four different types of models with their own transfer functions (detailed examples of each type and associated transfer function structures are given in Refs. 8 and 11). The model chosen for the purposes of this article is a cascade model with one of its ends constrained and which is being excited by the dynamic force. The choice is motivated by the close resemblance of such a model to a fixed piezoelectric stack, where individual plates can be described as successive stages of the model. According to the models proposed in Refs. 8 and 11, a hypothetical transfer function for an infinite degree cascade model constrained on one end and excited with a dynamic force takes the factorial form:

$$T\left(s\right)=H\frac{\left(s^{2}+\omega_{1}^{2}\right)\left(s^{2}+\omega_{3}^{2}\right)\ldots \left(s^{2}+\omega_{n-2}^{2}\right)\left(s^{2}+\omega_{n}^{2}\right)}{s\left(s^{2}+\omega_{2}^{2}\right)\left(s^{2}+\omega_{4}^{2}\right)\ldots \left(s^{2}+\omega_{n-1}^{2}\right)},$$ (1)

where:

*T*(s) – response of a system,

*H* – response amplification factor,

*ω* – consecutive frequencies (even are resonant and odd are anti-resonant),

*s* – Laplacian variable.

The factorial form is transformed into a standard polynomial form to define the coefficients for successive powers of the equation:

$$T\left(s\right)=H\frac{a_{i}s^{n}+a_{i-1}s^{n-2}+\ldots +a_{1}s^{2}+a_{0}}{b_{i}s^{n-1}+b_{i-1}s^{n-3}+\ldots +b_{1}s},$$ (2)

where:

*a*_*i*_ – numerator coefficients,

*b*_*i*_ – denominator coefficients.

After the initial calculation of the polynomial coefficients, the transfer function is converted into a continued fraction by using the Cauer’s method in its first form. ^17^ The method involves breaking down a polynomial into a chain fraction using simple mathematical operations. The first form of Cauer synthesis is implemented by dividing the coefficient of the greatest power of a numerator by the coefficient of the greatest power of a denominator. The resulting fraction is being multiplied by the denominator and subtracted from the polynomial function, leaving the rest, which is then inverted. This process is repeated until all powers of the transfer function are reduced:

$$I_{p}^{1}=\frac{a_{i}s^{n}}{b_{i}s^{n-1}}=\frac{a_{i}}{b_{i}}s,$$ (3)

$$R_{p}^{1}=\frac{a_{i}s^{n}+a_{i-1}s^{n-2}+\ldots +a_{1}s^{2}+a_{0}}{b_{i}s^{n-1}+b_{i-1}s^{n-3}+\ldots +b_{1}s}-I_{p}^{1},$$ (4)

$$R_{p}^{j+1}=\frac{1}{R_{p}^{j}}-I_{p}^{j+1},$$ (5)

where:

$I_{p}^{i}$ – fraction extracted by division of parameters next to the highest power “s”,

$R_{p}^{i}$ – rest of the original polynomial after subtraction.

A continued fraction resulting from the process can be used to determine the values of discrete component parameters in the synthesized system:

$$\frac{T\left(s\right)}{H}=I_{p}^{1}∙s+\frac{1}{I_{p}^{2}∙s+\cdots +\frac{1}{I_{p}^{n-1}∙s+I_{p}^{n}∙s}}.$$ (6)

**Figure 1.**
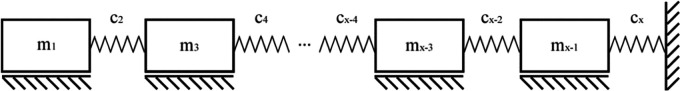
A mechanical cascade system composed of an infinite number of stages composed of mass “m” and spring “c” pairs, synthesized from the transfer function polynomial with the Cauer’s method.

Obtained values can be used in either mechanical or electrical cascade systems which gives this method a high degree of flexibility. An example of an infinite stage mechanical system resulting from the calculations have been shown in Figure 1.

The newly obtained mechanical model consists of springs and inertial components which can be used to determine the stiffness and mass/density of the piezoelectric material. Damping elements can be added to the model to account for energy dissipation which is an inherent property of piezoelectric materials. The mechanical quality factor is often used to indicate piezoelectric mechanical damping ability. The factor is inversely proportional to the amount of damping generated by the material and can be derived from Rayleigh’s method. ^28^^,^^31^ However, damping introduces complex derivatives into the equations of motion for any mechanical or electrical system. Every system can be considered under the case of subcritical, critical, and over-critical damping. Depending on the strength of damping elements, a system can be in a state of damped oscillations (subcritical damping) or the oscillations may stop immediately after the harmonic excitation disappears (critical damping), they do not occur (over-critical damping). Because of that, there are three separate integral solutions that describe each case respectively. A resonance (additive interference of harmonic oscillations) occurs only under the case of subcritical damping. In order to limit the range of possible solutions, it was assumed that all models would only be considered under subcritical damping, thus eliminating cases where vibrations cannot resonate. The decision was made because the project was focused on piezoelectric applications where vibration generation was their main purpose. Another assumption limited the range of solutions to those with a Rayleigh damping coefficient related to stiffness. According to literature, ^24^ most cases of piezoelectric damping are restricted to damping with respect to element stiffness only. The two assumptions simplify the term for the damping coefficient. Based on the obtained equations, it is then possible to determine the damping parameters for each stage in relation to the spring stiffness:

$$\beta <\frac{2}{\omega_{1}+\omega_{n}},$$ (7)

$$b_{i}=\beta c_{i},$$ (8)

where:

β – stiffness damping coefficient,

$b_{i}$ – damping value,

$c_{i}$ – spring stiffness.

**Figure 2.**
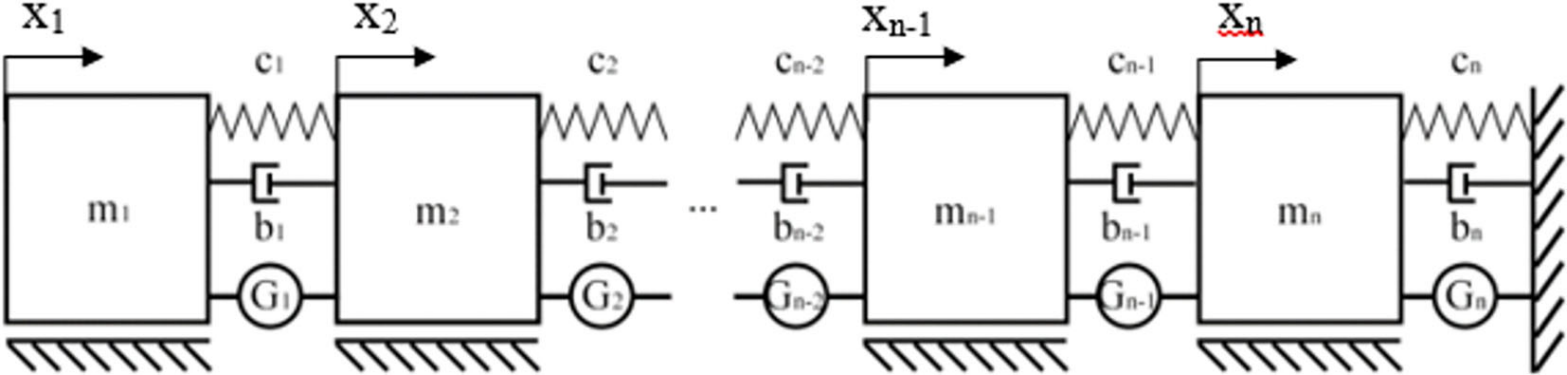
A discrete cascade mechanical model with added damping elements. The model masses are marked with “m” symbols, springs with “c” symbols, dampers with “b” symbols and electromotive forces with “G” symbols. The displacement of each mass was marked with “x”.

To verify the model in its current state, an intermediate electromotive force was also added at each stage. Its role was to simulate the actuation of piezoelectric plates by an electrical stimulus, similar to. ^15^^,^^25^ The electromotive force acts as a simplified force generated by the electromechanical coupling used to describe the conversion of energy conversion between the mechanical and electrical part of a piezoelectric plate. The mechanical model with added damping and electromotive forces is shown in Figure 2.

### Mechanical model verification

The synthesized model had to be verified after the addition of new damping elements and additional forces. An analysis of the model response was carried out in terms of its magnitude along the frequency axis. Two analysis methods were used to verify the existing mechanical model. The classical matrix method served as a reference for an additional method using structural numbers. ^2^^,^^11^^,^^16^^,^^29^ The use of a second method is considered as part of the ongoing research ^30^ aimed at evaluating the accuracy and computational load of structural numbers in comparison to the matrix calculation. Current state of the algorithm based on structural numbers should still not be considered as final as there are more improvements to be made in terms of its compatibility with computation software. Starting with the classical method, a set of equations of motion was created to describe relations between parts of the model. To avoid overcomplicating the example, only two degrees of freedom were considered. The equations of motion for a two-stage model derived from the Lagrangian equations were as follows:

$$\left\{\begin{array}{l} G_{1}=m_{1}{\ddot{x}}_{1}+b_{1}\left({\dot{x}}_{1}-{\dot{x}}_{2}\right)+c_{1}\left(x_{1}-x_{2}\right)\\ G_{2}=m_{2}{\ddot{x}}_{2}-b_{1}\left({\dot{x}}_{1}-{\dot{x}}_{2}\right)+b_{2}{\dot{x}}_{2}-c_{1}\left(x_{1}-x_{2}\right)+c_{2}x_{2} \end{array}\right..$$ (9)

Under the assumption into account, that the system is only considered in the case of subcritical damping, an integral of a second-order differential equation ^32^ for the variable displacement *x* can be described by the equation:

$$\left\{\begin{array}{l} x_{1}=A_{11}\text{sinωt}+A_{12}\text{cosωt}\\ x_{2}=A_{21}\text{sinωt}+A_{22}\text{cosωt} \end{array}\right.,$$ (10)

where:

$A_{i1}$ – real part of the *i* ^th^ element amplitude response,

$A_{i2}$ – imaginary part of the *i* ^th^ element amplitude response.

Combining (9) with (10) and separating the components with sine and cosine coefficients, then reordering the equations by combining all the coefficients standing next to consecutive amplitudes, yields a coefficient matrix:

$$\left[\begin{array}{cccc} k_{1}-m_{1}\omega^{2} & -b_{1}\omega & -k_{1} & b_{1}\omega \\ b_{1}\omega & k_{1}-m_{1}\omega^{2} & -b_{1}\omega & -k_{1}\\ -k_{1} & b_{1}\omega & -m_{2}\omega^{2}+k_{1}+k_{2} & -b_{1}\omega -b_{2}\omega \\ -b_{1}\omega & -k_{1} & b_{1}\omega +b_{2}\omega & -m_{2}\omega^{2}+k_{1}+k_{2} \end{array}\right].$$ (11)

Based on the obtained coefficient matrix (11) and a vector of actuating forces, a vector of amplitudes can be calculated. To obtain absolute displacement, the sine and cosine coefficient must be combined using Pythagoreans’ theorem:

$$\begin{array}{l} A_{1}=\sqrt{{\left(A_{11}\right)}^{2}+{\left(A_{12}\right)}^{2}}\\ A_{2}=\sqrt{{\left(A_{21}\right)}^{2}+{\left(A_{22}\right)}^{2}} \end{array}$$ (12)

To calculate phase-shift of each stage along the frequency spectrum, a tangent between the real and imaginary component of displacement can be calculated:

$$\begin{array}{l} \varphi_{1}=\text{atan}\frac{A_{11}}{A_{12}}\\ \varphi_{2}=\text{atan}\frac{A_{21}}{A_{22}} \end{array}.$$ (13)

The set of derived equations (12, 13) allows the amplitude response and phase shifts to be determined for any cascade system built using standard discrete elements representing model inertia, stiffness, and damping. Calculations are relatively quick and easy when considering systems with a small number of degrees of freedom. However, calculations become much more complex and computationally intensive for systems with a large number of degrees of freedom. This is an effect of the number of elements being multiplied in the determinant of a matrix. The numbers get big really fast because of the accumulation of the powers when all the matrix components are being combined. This problem prompted the search for alternative solutions for rapid analysis of more complex systems.

One of the alternatives to matrix calculations is being developed by the researchers at the Silesian University of Technology. ^2^^,^^7^^,^^8^^,^^11^^,^^16^^,^^29^ The non-classical method of system analysis involves the use graphs and structural numbers to illustrate relations between forces in a system. The structural number method offers an alternative solution for deriving equations of motion by using a graphical representation of relations in the system. Each edge of the graph corresponds to a single discrete relation between two elements of a system, represented by nodes. All the edges and nodes in the graph are assigned numbers called “structural numbers”. These numbers form structures similar to matrices based on edge connections with nearby nodes. Using the algebra of structural numbers, it is then possible to derive a system of equations similar to equations of motion. A detailed description of this method in the context of solving mechanical cascade systems without damping was presented in Ref. 30. The structural number algebra is an entirely separate method of performing mathematical operations and therefore requires extensive explanation. It is strongly recommended to consult the book of the original author of this method ^29^ for the details on its usage.

**Figure 3.**
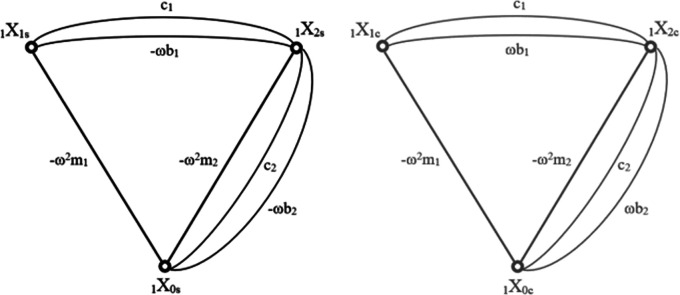
Graph of the cascade model where: a) shows the sine coefficients b) shows the cosine coefficients. The nodes of each graph represent the degrees of freedom of the model they are describing. Each edge corresponds to forces acting between each degree and is being described by the formula of elements creating each force. “ω” is the frequency, “m” is the mass of each element, “c” corresponds to spring loads and “b” to damping.

In the context of this article, several necessary changes had to be introduced to account for damping elements in the model. The basic method does not allow a simple decomposition of the sine and cosine coefficients when damping is present in the system. A standard approach includes resolving the equations with motion derivatives replaced with Laplacian transform. The issue arises when the final equation obtained with structural number algebra is turned back into its derivative form. When motion derivatives are replaced with the integral solution for sub critically damped motion as in (10), the equation becomes extremely tangled. To overcome this problem, an intermediate method has been developed, which realises the mentioned decomposition of sine and cosine components (10) prior to the application of the structural number algebra, Effectively, that means that the entire graph of relations was duplicated, separating the edges corresponding to sine and cosine coefficients. Each case is then being calculated separately until the last step. Both resulting graphs are shown in Figure 3.

The structural numbers of both graphs are synonymous, the only difference being the opposite sign in the first derivatives of the sine and cosine function, which relate to the damping coefficients. As with the matrix method, the sine graph corresponds to the real part of the calculation and the cosine part to the imaginary part. This time, in order to obtain absolute values of the displacements, the merging (similar to (12)) has to be performed for the whole structural number and all the simultaneity functions. ^11^^,^^29^^,^^30^ The amplitude response of each stage can be calculated by solving the equations:

A11=Simz∂Dω∂1G11∂Dω+Simz∂Dω∂2∂Dω∂1G21∂Dω, (14)

$$A_{1}=\sqrt{{\left(A_{11}\right)}^{2}+{\left(A_{12}\right)}^{2}},$$ (15)

$$D\left(\omega \right)=\sqrt{D_{s}{\left(\omega \right)}^{2}+D_{c}{\left(\omega \right)}^{2}},$$ (16)

where:

$D_{s}\left(\omega \right)$ – structural number for the sine graph,

$D_{c}\left(\omega \right)$ – structural number for the cosine graph,

$A_{\mathrm{ij}}$ – amplitude calculated with simultaneity function.

The process of obtaining the necessary structural numbers to derive equations (14– 16) and the meaning of a simultaneity function has been explained in more detail in. ^29^^,^^30^ The computer algorithm which was later used for this purpose is described in detail under the section dedicated to the algorithm testing.

### Electromechanical model synthesis

The synthesized mechanical model serves as a basis for the construction of a combined electromechanical model of a piezoelectric system. Constitutive equations and analogy between mechanical and electrical components were used to derive parameters of the piezoelectric model. ^14^^,^^19^^,^^24^^,^^25^ The electromechanical coupling coefficient for piezoelectric coupling perpendicular to the plate surface was determined based on resonant, anti-resonant frequency pairs:

$$k_{33}^{2}=\frac{\frac{\pi }{2}}{1+\frac{∆f}{f_{r}}}\tan\frac{\frac{\pi }{2}+\frac{∆f}{f_{r}}}{1+\frac{∆f}{f_{r}}},\;∆f=f_{a}-f_{r},$$ (17)

where:

$k_{33}$ – electromechanical coupling factor in the 3-3 direction,

$f_{a}$ – anti-resonant frequency of a given stage,

$f_{r}$ – resonant frequency of a given stage.

A broader term defining electromechanical coupling for a general case, called an effective coupling coefficient was used to approximate the capacity of piezoelectric plates ^33^:

$$\frac{k_{\mathrm{eff}}^{2}}{1-k_{\mathrm{eff}}^{2}}=\frac{∆f^{2}}{f_{r}^{2}},$$ (18)

$$C_{p}=\frac{k_{\mathrm{eff}}C_{m}}{1-k_{\mathrm{eff}}},$$ (19)

$$C_{m}=\frac{1}{c_{m}},$$ (20)

where:

$k_{\mathrm{eff}}$ – effective electromechanical coupling factor,

$C_{p}$ – plate capacitance,

$C_{m}$ – equivalent capacitance from mechanical stiffness,

$c_{m}$ – mechanical stiffness of a lumped piezoelectric model.

Another property of a piezoelectric material frequently mentioned in specifications is the mechanical quality factor. This property describes the amount of energy that is being lost during dynamic operation of a piezoelectric plate. It corresponds to mechanical losses in a system. An approximate value of a mechanical quality factor can be calculated using the equation derived from the established Rayleigh’s method:

$$Q_{m}=\frac{1}{\beta \omega }.$$ (21)

Electrical permittivity can be also approximated if piezoelectric capacitance is known by using an equation:

$$\varepsilon_{33}^{T}=\frac{C_{p}h}{A},$$ (22)

where:

$\varepsilon_{33}^{T}$ – electrical permittivity of a piezoelectric material in 3-3 direction under constant stress,

h – plate thickness,

A – plate cross section field.

Lastly, based on the lumped stiffness of the mechanical model, a piezoelectric material stiffness can be obtained using Hooke’s law:

$$c_{33}^{E}=c_{m}\frac{h}{A},$$ (23)

where:

$c_{33}^{E}$ – piezoelectric stiffness in 3-3 direction under constant electrical field.

### Piezoelectric discrete model verification

To analyse piezoelectric response with calculated parameters, a discrete electromechanical model was created based on the Butterworth-Van Dyke model and other lumped piezoelectric models available in the literature. ^13^^–^^15^^,^^34^ The basic form of constitutive equations for piezoelectric effect in a 33 mode was used:

D3=ε33TE3+d33T3S3=d33E3+s33ET3, (24)

where:

$D_{3}$ – electric displacement field,

$S_{3}$ – mechanical strain,

$E_{3}$ – electric field,

$T_{3}$ – mechanical stress,

$d_{33}$ – piezoelectric charge constant,

$s_{33}^{E}$ – mechanical compliance constant (inverse of mechanical stiffness constant).

The equation (24) was modified using Hooke’s law and by converting equations for underlying variables:

$$E_{3}=\frac{U_{p}}{h},$$ (25)

where: $U_{p}$ – voltage across the piezoelectric plate thickness,

$$D_{3}=\frac{Q_{p}}{A},$$ (26)

where: $Q_{p}$ – charge across the piezoelectric plate surface.

**Figure 4.**
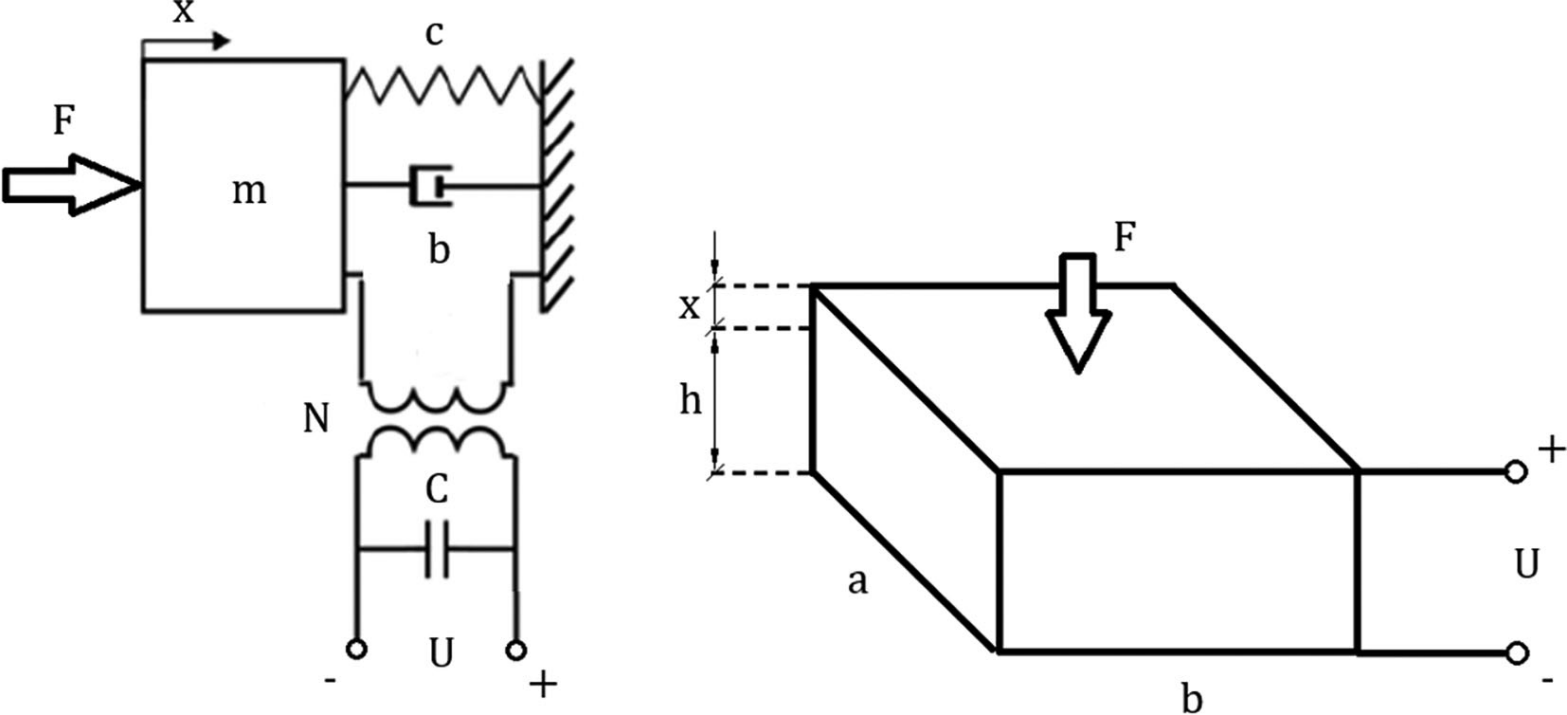
Discrete piezoelectric model composed of mechanical lumped model combined by a transformer with an electrical network. “F” represents an external force, “c” is the mechanical stiffness, “b” corresponds to damping, “N” is the electromechanical coupling, “C” is the electrical capacitance of the piezoelement and “U” represents the voltage exciting the circuit. “a,b,h” correspond to geometric dimensions of the piezoelectric element, while “x” represents the displacement.

By combining the constitutive equations with (25, 26) and plugging the equations of motion for the initial mechanical model (9) into the mechanical input of the electromechanical system, a lumped piezoelectric model was created. Relations inside a single piezoelectric plate are described by a system of equations:

mx¨+bx˙+cmx=−cmd33Up+FU0Cp=xcmd33+Upε33TAh1−k332, (27)

where:

F – external force applied to a piezoelectric plate,

$U_{0}$ – external voltage applied to a piezoelectric plate,

m – lumped piezoelectric mass,

b – lumped piezoelectric damping,

$c_{m}$ – lumped piezoelectric stiffness,

$U_{p}$ – lumped piezoelectric capacitance,

The discrete piezoelectric model has been shown in Figure 4. Inputs and outputs were extracted from (27) by grouping parameters and converting the equation into a Laplace form. The resulting matrix was used to create an algorithm for piezoelectric model analysis:

$$\left[\begin{array}{c} X\left(s\right)\\ U_{p}\left(s\right) \end{array}\right]∙\left[\begin{array}{cc} ms^{2}+\mathrm{bs}+c_{m} & c_{m}d_{33}\\ c_{m}d_{33} & \varepsilon_{33}^{T}\frac{A}{h}\left(1-k_{33}^{2}\right) \end{array}\right]=\left[\begin{array}{c} F\\ U_{0}C_{p} \end{array}\right].$$ (28)

The top left coefficient of the coefficient matrix (28) refers to purely mechanical transformations within the piezoelectric plate. Top right and bottom left coefficients refer to the electromechanical coupling. The bottom right coefficient refers to purely electrical transformations. The matrix can be easily expanded by adding more stages to the model as well as it can be calculated using the matrix method mentioned in (10).

### Algorithm testing

A series of computer programs were prepared to test the accuracy and computational load of used algorithms. All the programs were written using mathematical software called MATLAB in version R2019b (RRID:SCR_001622). The code was prepared using basic functions offered by the standard MATLAB suite.

The polynomial equation was created using “for” loops which added consecutive elements to its product form, depending on the number of frequencies given as an input. The MATLAB software operates interchangeably between the product form and exponential form of polynomials, so the conversion was done automatically. The Cauer synthesis was realised with basic program loops which continuously calculated the remainders of the polynomial function, as was explained with equations (3, 4, 5). Based on the order of elements in the resulting chain fraction, consecutive parameters in form of spring loads and masses were extracted from the equation in each iteration of the loop.

The analysis method using matrix calculations was realised by first creating the equations of motion (9) depending on the number of masses given as an input. The displacement was described using a substitute parameter “X” which then was replaced in bulk with derivatives of the sine cosine integral (10) by using MATLAB function “subs”. Next, the equations were transformed into a matrix form and solved with the inbuilt MATLAB solver. The input vector of external forces was used to manipulate the output amplitudes of the matrix formula.

The structural number method was constructed from ground up, using vector transformation operations inside MATLAB. The structural numbers were represented by matrices consisting of simple numbers corresponding to numbered graph edges. Each number was bound to a set of equations representing forces acting inside the system. The method of assigning numbers to equations was explained in. ^30^ The structural number was formed by adding vectors (each vector consists of numbers corresponding to edges connected to a single graph vertex). The process was done using inbuilt MATLAB function called “combvec”. After that, several sorting operations were done in a loop to eliminate any repeats in the matrix representing the graph structural number. Further steps done to determine all simultaneity functions were also done by appropriately sorting and pruning the structural number matrix to create each structural number operation. To obtain final equations from the graph algebra, an intermediate step was introduced, which replaced each number with the equation that was bound to it. The step was also done using the “subs” function.

The eletromechanical system analysis was an extended matrix algorithm that was based on the equation (27). No new functions were introduced to make the algorithms and every step was made in analogy to the matrix method used to analyse purely mechanical systems.

The code has been saved in a proprietary MATLAB format “.mlx” and placed in repositories of Mendeley Data site. ^35^^,^^36^ The code placed in the repository ^35^ contains algorithms used for the synthesis of mechanical systems and their comparison through the matrix and structural number methods. The repository ^36^ contains the algorithm used to synthesise and analyse a full eletromechanical model, simulating the real behaviour of a piezoelectric module. MATLAB is using an open standard for their file formats, so it is possible to open files using standard Windows tools. For reproduction purposes a user has to convert the extension of the file format into “.zip” and open its contents through the “.xml” file stored inside the package. It must be noted however, that the precision and calculation times may vary, depending on the type of software used to reproduce the results and even on the hardware used to process the computation tasks. It is also necessary to note that some of the code may have to be rewritten because of the use of MATLAB specific syntax that may be different in other software. For the sake of consistency and repeatability, all calculations were done on a single computer with a Ryzen 7 2700X processor and a 4 × 8 GB kit of DDR4 CL16 RAM clocked at 3000 Mhz.

Algorithms used to compare the precision and computation time of both matrix and structural number methods were published in an on-line repository. ^35^ To compare the precision of both algorithms the chosen input data consisted of a collection of resonant (41 kHz and 55 kHz) and anti-resonant (47 kHz) frequencies. The frequencies were chosen arbitrarily based around a rough estimation of piezoelectric elements resonating at the lower spectrum of the possible range of resonating piezoelectric modules. The necessary system component parameters were calculated using the synthesis algorithm. The damping ratio was left at 100% and the scaling factor *H* was set to a value of 0.01. The dynamical forces were set to 1N of harmonic force with a cosine wave on both degrees of freedom. The program named “comparison” ^35^ was used to compare the precision of both algorithms and produce the resulting graphs and calculate deviations for the extrema.

A simple test was also carried out to check how computationally demanding are both methods in comparison to each other. For that purpose, two programs called “Matrix_method” and “Structural_number_method” were written in MATLAB R2019b. Both algorithms were written with a fixed set of model parameters and a fixed set of equations. To avoid long computation times, parameters were chosen for a two-degree system resonating at frequencies of 10 rad/s and 22 rad/s with an antiresonant frequency of 17 rad/s. The computation time was measured for both methods using the inbuilt MATLAB timer function “tic/toc”. The timer measured seconds that the program took to form the equation and calculate the amplitudes in both cases. Each program was run 10 times with the exact same settings and all calculation time measurements were noted down with an average time derived additionally. Before each run, all the variables generated in the previous runs were cleared to avoid any potential changes in computation speed because of reusing the stored results.

For the analysis of the electromechanical system, model with a single degree of freedom was chosen. The resonant frequency was 590kHz and the scaling factor *H* was set to 0.0025. The damping factor *β* was calculated from an existing mechanical quality factor *Q _m_* of 83 using equation (7) which was inversed. The remaining input parameters such as plate dimensions, plate stiffness and capacitance were either taken from an existing piezoelectric material specification or derived using the known physical properties (17-26) ( http://www.steminc.com/piezo/PZ_property.asp). All parameters used in the case of this article are also contained in the program called “analysis_piezoelectric_system” in the on-line repository. ^36^

## Results

**Figure 5.**
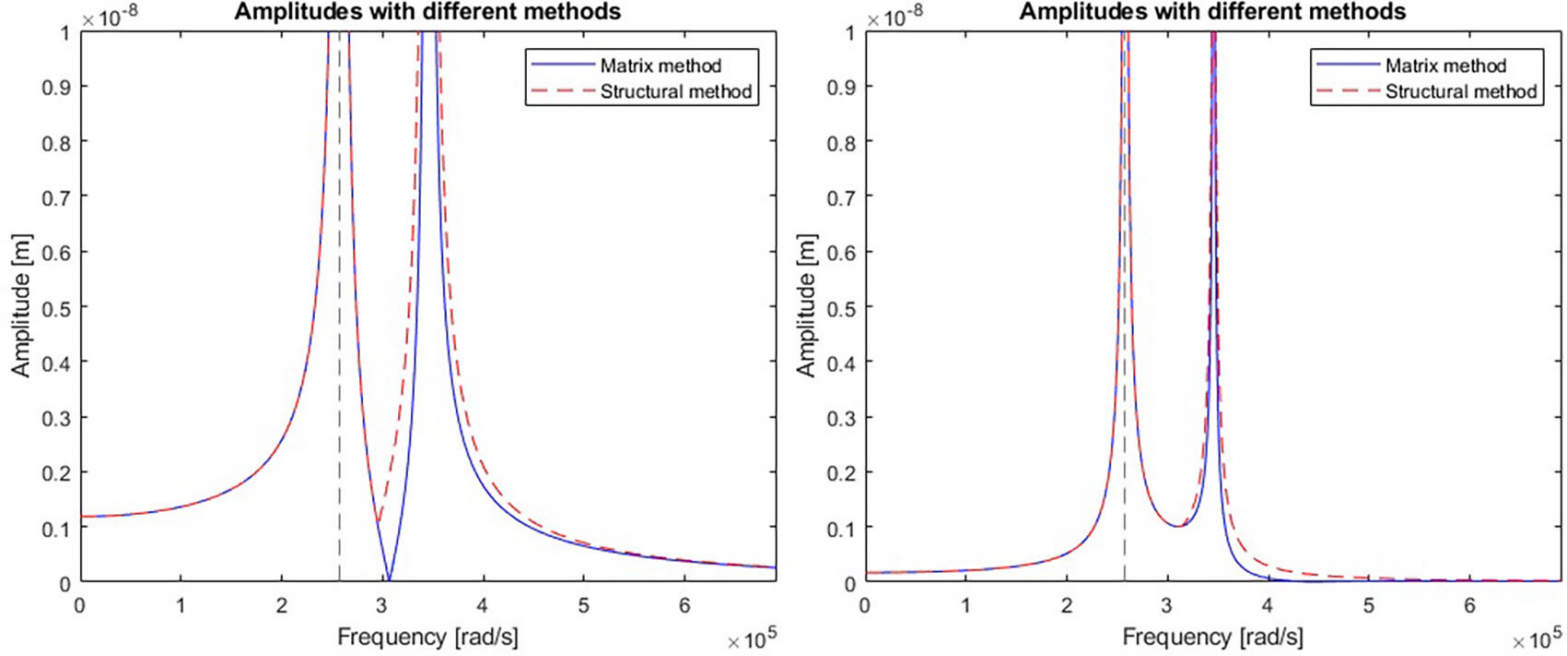
Model response calculated for a) first vibrating mass, and b) second vibrating mass. Blue line represents a response calculated with matrix method; red line represents the response calculated with structural number algebra.

A comparison of the efficiency and accuracy of the two methods was done with the comparing program. A two-stage mechanical system with two resonant frequencies of 41kHz and 55kHz (257611rad/s and 345575rad/s respectively) was used for the comparison. The graphs in Figure 5 show the displacement amplitudes for both degrees of freedom of the considered case, obtained by matrix calculation and structural numbers.

It is clear that there are some differences between the results obtained by the two methods. To gain a better understanding, derivatives were taken from the model response equations to calculate the local extremes obtained by both functions. Table 1 gives a direct and relative comparison between the frequencies obtained with both functions. Table 2 gives the calculated values of the displacement amplitude for each characteristic frequency.

**Table 1.** Comparison of frequencies between model responses obtained with both methods.

	Matrix method	Structural numbers	Direct difference	Indirect difference
**1** ^**st**^ **stage**	257611 rad/s	257611 rad/s	0 rad/s	0%
	345575 rad/s	345575 rad/s	0 rad/s	0%
**2** ^**nd**^ **stage**	257611 rad/s	257611 rad/s	0 rad/s	0%
	345575 rad/s	345575 rad/s	0 rad/s	0%

**Table 2.** Comparison of amplitude responses obtained with both methods on the nodes.

	Matrix method	Structural numbers	Direct difference	Indirect difference
**1** ^**st**^ **stage**	0.0101 mm	0.0101 mm	0 mm	0%
	0.0017 mm	0.0027 mm	0.001 mm	58.82%
**2** ^**nd**^ **stage**	0.0033 mm	0.0033 mm	0 mm	0%
	0.0004 mm	0.0006 mm	0.0002 mm	50%

The nodal frequencies match perfectly, but there are significant differences in the amplitudes obtained around the second resonant frequency of the system in both stages. The alternative method using structural numbers is sufficient if the priority is only to check the nodal frequencies. However, when it comes to accuracy in terms of amplitude response, this method is subject to considerable error.

Another problem with the structural number method is that the authors were unable to establish the equations necessary to calculate the phase shifts in the system’s response.

Table 3 shows the computation time and an average time from all measurements done on both algorithms based on matrix and structural number methods. ^35^ The test showed that matrix method took an average of 13.972 s to calculate the amplitude response of a two-stage system. The alternative method took only 8.637 s on average, which is 38% faster than the classical method.

**Table 3.** Computation time for both methods used to analyse a simple mechanical system.

Trial	Matrix method	Structural numbers
**1**	12.815 s	7.531 s
**2**	12.824 s	7.649 s
**3**	12.761 s	7.577 s
**4**	12.759 s	7.546 s
**5**	13.099 s	7.563 s
**6**	12.905 s	7.578 s
**7**	12.839 s	7.626 s
**8**	13.162 s	7.574 s
**9**	13.080 s	7.570 s
**10**	13.088 s	7.627 s
**Average**	**12.933 s**	**7.584 s**

**Figure 6.**
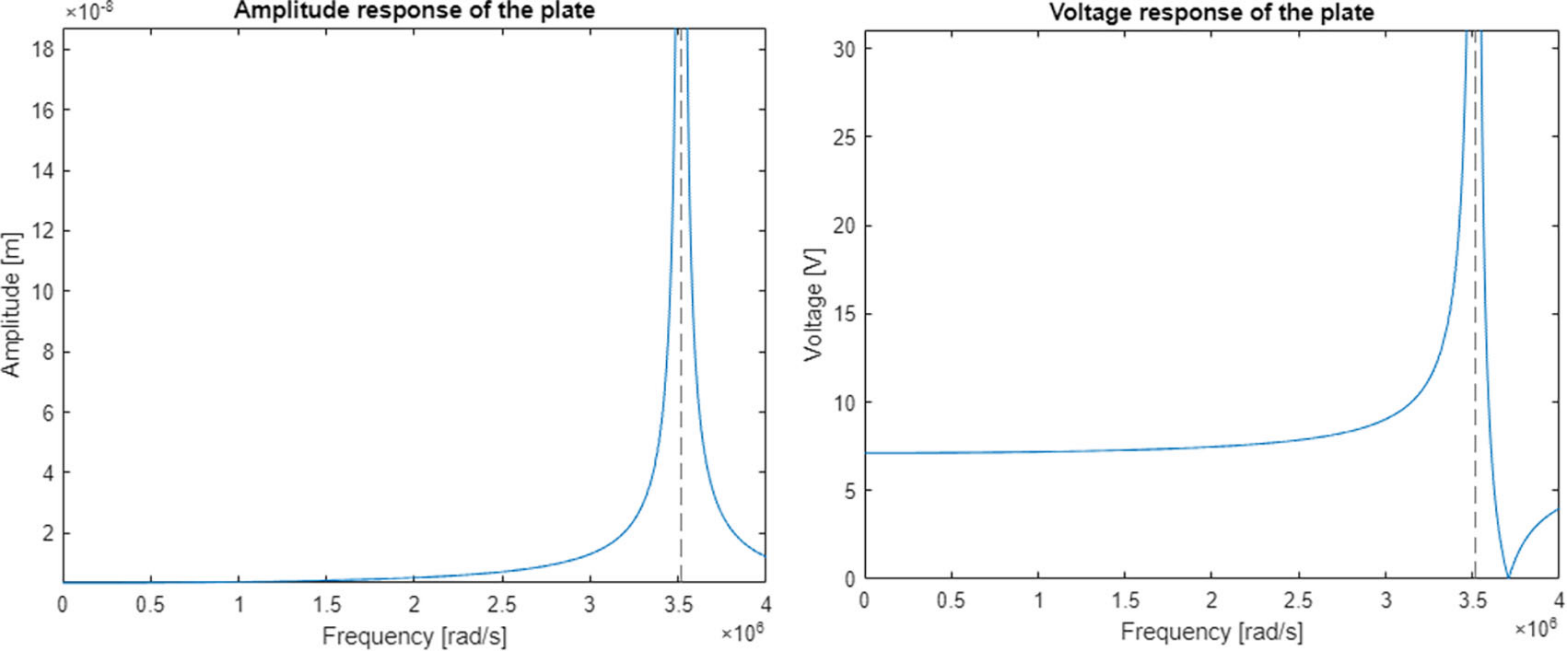
Model response calculated for a) displacement curve, and b) voltage curve along the frequency axis.

The discrete electromechanical model ^36^ was verified on a system simulating the behaviour of a single piezoelectric plate with specifications very close to the real ones taken from ( http://www.steminc.com/piezo/PZ_property.asp). The plate had to vibrate at a resonance frequency of 590kHz (3707079rad/s). Mechanical parameters of the plate were determined with the Cauer synthesis method. Piezoelectric plate dimensions were taken from ( https://www.steminc.com/PZT/EN/piezoelectric-plate-45x45x35mm-55-khz). Resulting graph of an amplitude response for piezoelectric plate displacement and voltage were shown in Figure 6.

The model had a resonance near the frequency of 560 kHz (3516624 rad/s) which gives an error of 5% in relation to the resonance target. The calculated magnitude of displacement at the resonant frequency is 2.6 mm which is well above the capability of a 3.5 mm thick piezoelectric plate. The voltage shows a significant increase in piezoelectric voltage in the resonant region and a complete reduction near the frequency of 590 kHz which was supposed to be the original resonant frequency.

## Conclusions

The aim of this work was to demonstrate a method that allows a piezoelectric model to be synthesized using only on a set of resonant/anti-resonant frequencies as the input data. The paper has shown that combining Cauer’s method of synthesis for mechanical or electrical networks with Rayleigh’s damping approximation and a variant of the Van Dyke-Butterworth model can yield a discrete electromechanical cascade model which parameters can be approximated to simulate the behaviour of a real piezoelectric component. This method differs from most other methods found in the literature ^13^^–^^15^^,^^19^^,^^24^ in that it focuses on developing a new set of possible solutions based on a very limited information, rather than trying to break down a specific case under consideration. The ultimate goal of this method, which is under constant development, is to provide the ability to synthesize piezoelectric systems capable of resonating in a defined range of frequencies required for a target solution. An example application of such a method could be an attempt to scale up the active vibration cancellation technology to dampen vibrations generated by motors and engines. This technology is currently used in many audio solutions and new, larger piezoelectric resonators could be used for more industrial oriented projects. This method offers the possibility of constructing a piezoelectric stack of plates which have different material properties or geometrical sizes to create multiple resonant frequencies within a single module.

The method presented in this article relies heavily on mechanical model synthesis in its initial stage. In order to proceed with confidence, it was necessary to verify the mechanical model before using it in later stages of the work. However, for more complex systems the classical matrix calculation creates a significant computational load, which has the effect of significantly extending the computation time. An additional set goal was to develop an alternative method for mechanical system analysis using graphs and structural numbers. The presented alternative solution gave promising results in terms of computation time. The accuracy of calculated characteristic frequencies between the standard matrix method and the proposed alternative using structural numbers was on par in terms of calculated peaks near resonant frequencies. However, the calculated amplitudes differed significantly from those calculated using the matrix method, used as a reference. The issue was tested on different models and the inconsistency of calculated responses was random. In its current state the algorithm can be useful for verifying only the resonant frequencies of a model. This method definitely needs further fine-tuning and testing to correct the errors in the amplitudes obtained and to further verify the advantages indicated.

The final verification of the piezoelectric model has shown a small deviation from the target resonant frequency. An initial hypothesis is that this error could be corrected by applying a correction factor to the input frequency. A bigger set of tests on the algorithm has to be conducted to verify it. Another problem highlighted was the calculated amplitude response for the resonant frequency which was several orders of magnitude greater than the expected value of displacement for a plate of given thickness. The cause of this problem is currently unknown. It is suspected that the problem may be due to an error in the algorithm, or it may be caused by differences between the real piezoelectric parameters for a plate that is vibrating at its resonant frequency and its specification which is measured for a frequency of 1kHz. It is clear that this model needs more tests before it can be considered acceptably accurate. The reference plate specification (more information here) also didn’t contain enough data to reliably compare the results obtained from the model analysis. Only a rough comparison of material properties could be conducted. The data of specific plates such as their exact resonant or anti-resonant frequencies was sometimes missing as well as information about their intended mode of vibration (longitudinal, thickness wise) and related coupling factors. It is necessary to plan a series of empirical tests to obtain reference data more reliably for model validation.

## Data Availability

Reference data for the specific piezoelectric module has been sourced from a piezoelectric materials vendor: https://www.steminc.com/PZT/EN/piezoelectric-plate-45x45x35mm-55-khz. A list of detailed material properties used for algorithms and the electromechanical model analysis was taken from: http://www.steminc.com/piezo/PZ_property.asp. Mendeley Data: Model algorithms. https://doi.org/10.17632/gdt8yr9m3d.4 ^35^ This project contains the following data:
-
Comp_time_test.xlsx -
Comparison_value_set.mat Mendeley Data: Piezoelectric electromechanical model (first iteration), https://doi.org/10.17632/pydnfmkfdk.3 ^36^ This project contains the following underlying data:
- electromech_model.mat Data are available under the terms of the Creative Commons Zero “No rights reserved” data waiver (CC0 1.0 Public domain dedication).

## Software availability

### Model algorithms

Source code available from:

Archived source code available from: https://doi.org/10.17632/gdt8yr9m3d.4 ^35^

License: CC0 1.0

Piezoelectric electromechanical model

Source code available from:

Archived source code available from: https://doi.org/10.17632/pydnfmkfdk.3 ^36^

License: CC0 1.0
