# Supplementary material for: Analytical approach to piezoelectric model synthesis with the use of Cauer’s method for system design
Source: F1000Res. 2024 Sep 6;12:1273. Originally published 2023 Oct 5. [Version 3] doi: 10.12688/f1000research.140943.3 (PMC11384192; doi:10.12688/f1000research.140943.3)
Supplement: Peer review history

# Reviewer report: Reviewer response for version 3

**DOI:** 10.5256/f1000research.170982.r321408

**Published:** 2024-09-09

**Authors:** Pedro M. Ferreira

**Affiliations:** 1 Universidade Nova de Lisboa, Lisbon, Lisbon, Portugal

All the comments have been taken into account, the manuscript is in a position to be approved.

Is the work clearly and accurately presented and does it cite the current literature?

Partly

If applicable, is the statistical analysis and its interpretation appropriate?

Not applicable

Are all the source data underlying the results available to ensure full reproducibility?

Yes

Is the study design appropriate and is the work technically sound?

Yes

Are the conclusions drawn adequately supported by the results?

Partly

Are sufficient details of methods and analysis provided to allow replication by others?

Yes

Reviewer Expertise:

Piezoelectric Composites, Piezoelectric Behaviour, Advanced Materials, Smart Materials

I confirm that I have read this submission and believe that I have an appropriate level of expertise to confirm that it is of an acceptable scientific standard.

# Reviewer report: Reviewer response for version 3

**DOI:** 10.5256/f1000research.170982.r321409

**Published:** 2024-09-09

**Authors:** Guangtao Lu

**Affiliations:** 1 Wuhan University of Science and Technology, Wuhan, Hubei, China

The manuscript has been greatly improved, and the authors have responsed to all the main concerns. As the authors replied to comments, they are still trying their best to further investigate and solve some main concerns the two reviewers point out. Of course, I am looking forward to your newest study.

Is the work clearly and accurately presented and does it cite the current literature?

Yes

If applicable, is the statistical analysis and its interpretation appropriate?

Not applicable

Are all the source data underlying the results available to ensure full reproducibility?

Yes

Is the study design appropriate and is the work technically sound?

Partly

Are the conclusions drawn adequately supported by the results?

Yes

Are sufficient details of methods and analysis provided to allow replication by others?

Partly

Reviewer Expertise:

Structural health monitoring by uisng lamb waves

I confirm that I have read this submission and believe that I have an appropriate level of expertise to confirm that it is of an acceptable scientific standard.

# Reviewer report: Reviewer response for version 2

**DOI:** 10.5256/f1000research.164274.r261934

**Published:** 2024-08-28

**Authors:** Guangtao Lu

**Affiliations:** 1 Wuhan University of Science and Technology, Wuhan, Hubei, China

Thank the authors for their response and detailed revisions. The current version has been greatly improved, and however there are still some concerns that need be addressed. Moreover, impressed by the innovative effort.

1. If the name of the proposed method is included in the title, the novelty of this manuscript will be more easily got by the readers.
2. Figure 3: what do these symbols, _1_X _0s_, _1_X _1s_ and so on, stand for?
3. The material type and some key parameters of PZT should be clearly addressed instead of just putting a website link.
4. The resolution of the figures is low and their quality should be further improved.
5. Table 2 and 5: how to confirm the accuracy of the data?
6. Based on the results, the proposed method can be used to analyze the characteristics of piezoelectric systems accurately. However, the computation cost is much larger than the traditional method. Therefore, what is the merit or advantage of this method?

Is the work clearly and accurately presented and does it cite the current literature?

Yes

If applicable, is the statistical analysis and its interpretation appropriate?

Not applicable

Are all the source data underlying the results available to ensure full reproducibility?

Yes

Is the study design appropriate and is the work technically sound?

Partly

Are the conclusions drawn adequately supported by the results?

Yes

Are sufficient details of methods and analysis provided to allow replication by others?

Partly

Reviewer Expertise:

Structural health monitoring by uisng lamb waves

I confirm that I have read this submission and believe that I have an appropriate level of expertise to confirm that it is of an acceptable scientific standard, however I have significant reservations, as outlined above.

# Reviewer report: Reviewer response for version 2

**DOI:** 10.5256/f1000research.164274.r307986

**Published:** 2024-08-09

**Authors:** Pedro M. Ferreira

**Affiliations:** 1 Universidade Nova de Lisboa, Lisbon, Lisbon, Portugal

The manuscript presents a novel approach to synthesizing piezoelectric systems, starting with only known resonant and anti-resonant frequencies. This method offers a broader perspective on system modelling utilizing an algorithm based on the Cauer synthesis method, an initial discrete mechanical model is constructed, forming the foundation for piezoelectric model synthesis. The manuscript is well-composed and systematically organized. However, there are a few concerns and suggestions that should be addressed before it can be accepted for indexing. In my view, these issues require more detailed clarification before the manuscript can be considered for indexing.

1. The introduction to the present literature review is poor, particularly in the following excerpt: "Researchers largely focus on modelling simple piezoelectric components with lumped models and matrix tensor equations or try to approach the problem using FEM (finite element method) analysis." There are several models that could be referenced in the introduction, such as Eshelby-Type Models, Micromechanics-based models, or Asymptotic Homogenization-Based Models, which are widely used to model the behaviour of piezoelectric composites. Additionally, this excerpt needs to be supported with more current references. Keywords that could help in finding up-to-date references include: "Piezoelectric composites, Electro-mechanical properties, Analytical models, Numerical models."
2. In the last paragraph of the introduction, the excerpt “The number of rows and columns, however, is tightly connected to the shape of an edge graph which a given structural number relates to,” is very unclear. It should be rewritten for better clarity and explained in more detail.
3. The author compares the computation time and memory usage of parameterized algorithms, highlighting a significant increase in both when using the structural number method compared to the classical matrix method (as shown in Table 4). The authors should clarify why the calculation time and memory usage of the modified algorithms increase so dramatically as the number of degrees of freedom in the analysed systems rises.
4. The conclusion is overly long and needs to be more concise, with an emphasis on the limitations of the authors' analytical approach. For better clarity, consider presenting the main conclusions and limitations as bullet points.

Is the work clearly and accurately presented and does it cite the current literature?

Partly

If applicable, is the statistical analysis and its interpretation appropriate?

Not applicable

Are all the source data underlying the results available to ensure full reproducibility?

Yes

Is the study design appropriate and is the work technically sound?

Yes

Are the conclusions drawn adequately supported by the results?

Partly

Are sufficient details of methods and analysis provided to allow replication by others?

Yes

Reviewer Expertise:

Piezoelectric Composites, Piezoelectric Behaviour, Advanced Materials, Smart Materials

I confirm that I have read this submission and believe that I have an appropriate level of expertise to confirm that it is of an acceptable scientific standard, however I have significant reservations, as outlined above.

# Response

**Published:** 2024-08-23

**Authors:** Jacek Harazin

**Affiliations:** Politechnika Śląska, Poland

Thank You for your insightful review. In line with Your remarks, I have rewritten most of the introductory section of the article, adding more up-to-date literature references and including links to the numerical methods mentioned in the review. I also rewrote the research gap statement to make it more readable. The conclusion section has also been revised. Below I have included corresponding point by point responses to the various comments made in the review.

 1. *After rereading the introduction section, I must agree that it was too vague and contained too much information that could be considered irrelevant in the scope of the article. To amend that, I have completely rewritten most of this section, while also adding mentions and new references to current works (2020-2023) related with Eshelby-Type/micromechanical models. I must admit that I was so focused on the mathematical synthesis aspect in the context of piezoelectric models that I didn’t encounter works on models mentioned in your review. Thank You for bringing this issue up because that also helped me in finding new potential paths to expand my research in the future.*

 2. *I have decided to extend the explanation with a graphical clue in form of a new figure 1, which shows an example of a graph, from which a new structural number is being created. The example of a structural number has also been placed under new equation 1. I think that a simple figure might provide much more insight than my extensive explanations, while also conserving more space. In hindsight, I should have provided a descriptive figure in the first place.*

 3. *I agree that this issue was left with a vague explanation. It was already pointed out that the conclusions were overly long and realising that I was trying to make the explanation concise. Unfortunately, I made it too vague instead. I have tried to amend this by adding more detailed explanation in the conclusions. To summarize the explanation, the uncovered issue may be due to the structure of the written code for the method based on structural number algebra. It was made using closed loops which were combined with quite complicated substitution operations and complex algebraic representations of each graph edge. Combined, these three elements could contribute to the perceived great increase in both computation time and RAM requirement. The matrix method was largely an unmodified module readily available as an integral part of the Matlab software which may have been the reason for its much better performance. The new method had to be written from ground up which probably left a lot of the performance on the table due to my lack of programming skill and also the inability to better access program’s memory and CPU handling operations.*

 4. *Most of the conclusions section was reworked according to raised issues. I have tried to form several bullet points to streamline the thought process and make the text more accessible to readers. I wasn’t able to put the entire section in a set of bullet points, but I made an attempt to better separate the conclusions from both experiments described in the article to avoid confusion. I hope that will satisfy the requirements.*

# Reviewer report: Reviewer response for version 1

**DOI:** 10.5256/f1000research.154351.r222666

**Published:** 2023-11-29

**Authors:** Saurabh K. Yadav

**Affiliations:** 1 Institute of Infrastructure Technology Research and Management, Ahmedabad, Gujarat, India

The manuscript proposes a new approach for parameter estimation of piezoelectric structures based on the Cauer’s method, and compares its computational performance with the traditional matrix method. The authors claim that their method can synthesize a piezoelectric model using only a set of resonant and anti-resonant frequencies as the input data, and approximate the piezoelectric parameters using the synthesized mechanical model and some physical equations. The authors also use a non-classical method based on structural numbers and edge graphs to analyze the mechanical model and reduce the computational load. The manuscript is well-written and organized, and the mathematical derivations are clear and rigorous. However, I have some concerns and suggestions that need to be addressed before the manuscript can be accepted for publication.

- The main merit and novelty of the manuscript are not clearly stated in the abstract, introduction and conclusion. The authors should highlight how their method differs from the existing methods in the literature, and what are the advantages and limitations of their method.
- The authors use a single piezoelectric plate as an example to verify their electromechanical model. However, the results show that the model has a significant error in the resonant frequency and the displacement amplitude. The authors should explain the sources of this error and how it can be reduced or corrected. Moreover, the authors should test their model on more realistic and complex piezoelectric structures, such as stacks, rings, beams, etc., and compare their results with experimental data or other numerical methods.
- The authors compare the computational performance of their method with the matrix method using a two-degree system. However, this is not a fair comparison, as the matrix method can handle systems with any number of degrees of freedom, while the structural number method is limited by the complexity of the edge graph. The authors should compare the computational performance of both methods on systems with different numbers of degrees of freedom, and use more appropriate metrics, such as the number of floating point operations, the memory usage, the accuracy, etc.
- The authors use some references that are published in non-English languages, such as Refs. 8, 11 and 29. The authors should provide English translations of these references, or use more accessible sources that are published in English. The authors should also update their literature review with some recent works on piezoelectric modelling and parameter estimation.

Is the work clearly and accurately presented and does it cite the current literature?

Yes

If applicable, is the statistical analysis and its interpretation appropriate?

Yes

Are all the source data underlying the results available to ensure full reproducibility?

Yes

Is the study design appropriate and is the work technically sound?

Yes

Are the conclusions drawn adequately supported by the results?

Yes

Are sufficient details of methods and analysis provided to allow replication by others?

Yes

Reviewer Expertise:

Mathematical modelling

I confirm that I have read this submission and believe that I have an appropriate level of expertise to confirm that it is of an acceptable scientific standard, however I have significant reservations, as outlined above.

# Response

**Published:** 2024-03-25

**Authors:** Jacek Harazin

**Affiliations:** Politechnika Śląska, Poland

Thank you for the critical review. I would also like to apologise for a long break before my reply. I have prepared several corrections to the article regarding your review and they will be published with the new version. I've also tried to address your concerns directly:

 1. Let me repeat my answer to the second reviewer here, as it addresses the same concerns. The article showcases a new method to synthesize cascading electromechanical models using the Cauer method coupled with Van Dyke-Butterworth models to represent piezoelectric stacks with clumped mechanical and electrical components. Each of those methods have been already used by researchers but i have found no literature on the particular approach presented in this article. The novelty revolves around the general approach to synthesis with very limited input information. Most researchers focus on specific applications where piezoelectric parameters and the analysed structure is largely known or predetermined experimentally. Here, only the frequencies are known and there is an infinite number of possible models that could realise those predetermined resonant frequencies. Coupled with further steps presented in the article, this method offers a possibility to design piezoelectric stacks with different material properties or geometric dimensions, capable of resonating in predetermined frequencies.

 The limitation of this method is that it can only be used to synthesize models composed of components connected in series. So far, it can only be used to synthesise specific piezoelectric plate configuraions, where each component in a stack is responsible exclusively for a single resonant frequency set as the input. I have modified the article to better reflect those statements.

 2. Following up from my response to the second reviever, I agree and i can already attest that the method is currently being verified empirically. The synthesis method has been verified already with three real piezoelectric stacks with different geometrical dimensions which has shown some discrepancy between model results and real plate behaviour. The source of those differences was traced back to how some of the piezoelectric parameters are determined in the factory (under 1kHz wave stimulation) versus plate properties in the resonance proximity. It has to be noted as well, that this method relies on a lumped representation of an otherwise continous system. The main issue of this approach is that the lumped parameters are approximations of the real parameters and an electromechanical model currently can't perfectly match the behavior of a piezoelectric plate, where the interactions and their magnitude changes between the layers of a single piezoelement. There are also boundaries between piezoelectric elements, made with adhesives and isolating layers, which also introduce many hidden variables in the real model behaviour. The presented method is still in its infancy and requires more testing and refinement. I would like to provide a consice report on the empirical results in a follow up article though, as including all the information about the procedure and test stand would make this article very bloated.

 3. I was made aware that a comparison of calculation time used exclusively is a bad metric for algorithm complexity. I have tried looking for more literature on the matter and added more references to the article. Given my abilities and available resources, i have prepared a second study which focused more on the memory criterion. I've used the available Matlab evaluation modules to estimate the average RAM usage between both algorithms on a variety of models, along with a comparison between the average time it took both algorithms to complete. New suite of tests has uncovered more information about the structural number algorithm optimisation and shed some light on its shortcomings. I have modified the manusript to showcase the second study. I am aware that this method may also not be the best way to evaluate algorithm complexity and only presents a rough estimation and i will try to consult a specialist on the topic with an intent of providing some collaboration with more indepth information about algorithm complexity of the proposed method. 

 4. I have provided additional literature references to publications of the authors involved in the development of the structural number method where they descibe mentioned models in english. I hope that will provide the necessary background which was lacking previously.

 I hope that my response provides an exhaustive explanation to the issues raised.

 Best Regards,

# Reviewer report: Reviewer response for version 1

**DOI:** 10.5256/f1000research.154351.r222661

**Published:** 2023-11-29

**Authors:** Guangtao Lu

**Affiliations:** 1 Wuhan University of Science and Technology, Wuhan, Hubei, China

This manuscript proposed a new approach for parameter estimation of piezoelectric structures based on the Cauer’s method, and its computational performance is compared with the traditional method. However, in my opinion, some concerns need to be addressed more clearly before it is considered for publication.

1. What’s the main merit of the manuscript? It should be clearly addressed in the abstract, introduction and conclusion.
2. To compare the computational performance of the new model, the model response and computation time are compared with the matrix method. What are the errors of these two models? The two frequencies of the new model are compared with the ones of matrix method. Are the results of the matrix method accurate? What are the actual frequencies?
  It is the same during the amplitude comparison. Moreover, to compare the computational performance of the algorithm, there are many metrics, such as time cost, the number of floating points (FLOPs) multiple adds (MAdds), and so on. In addition, what is the meaning of only decreasing the computation time?
3. Page 4: “A combination of those factors creates four different types of models…” It refers to Refs. 8 and 11. However, Refs. 8 and 11 are published in non-English. Therefore, the details of the four models are suggested to be introduced in the manuscript.
4. Experimental results: the results should be further analyzed.

Is the work clearly and accurately presented and does it cite the current literature?

Yes

If applicable, is the statistical analysis and its interpretation appropriate?

Not applicable

Are all the source data underlying the results available to ensure full reproducibility?

Yes

Is the study design appropriate and is the work technically sound?

Partly

Are the conclusions drawn adequately supported by the results?

Yes

Are sufficient details of methods and analysis provided to allow replication by others?

Partly

Reviewer Expertise:

Structural health monitoring by uisng lamb waves

I confirm that I have read this submission and believe that I have an appropriate level of expertise to confirm that it is of an acceptable scientific standard, however I have significant reservations, as outlined above.

# Response

**Published:** 2024-03-25

**Authors:** Jacek Harazin

**Affiliations:** Politechnika Śląska, Poland

Thank you very much for the critical review and Your suggestions regarding the article clarity. I would also like to apologise for a long break before my reply. I have prepared several corrections to the article regarding your review and they will be published with the new version. I've also tried to address your querries to the best of my ability:

 1. The article showcases a new method to synthesize cascading electromechanical models using the Cauer method coupled with Van Dyke-Butterworth models to represent piezoelectric stacks with clumped mechanical and electrical components. Each of those methods have been already used by researchers but i have found no literature on the particular approach presented in this article. The novelty revolves around the general approach to synthesis with very limited input information. Most researchers focus on specific applications where piezoelectric parameters and the analysed structure is largely known or predetermined experimentally. Here, only the frequencies are known and there is an infinite number of possible models that could realise those predetermined resonant frequencies. Coupled with further steps presented in the article, this method offers a possibility to design piezoelectric stacks with different material properties or geometric dimensions, capable of resonating in predetermined frequencies.

 2. The predetermined frequencies and amplitudes were chosen arbitrarily and do not reflect any real piezoelectric system. A comparison between both analysis methods was done on the same model synthesized with the Cauer method. The matrix method was considered as a reference method because its a known and established way to calculate model response. The structural number method was compared against it and it was a pure comparison between two analytical methods.

 I was also made aware that a comparison of calculation time used exclusively is a bad metric for algorithm complexity. I have tried looking for more literature on the matter and added more references to the article. Given my abilities and available resources, i have prepared a second study which focused more on the memory criterion. I've used the available Matlab evaluation modules to estimate the average RAM usage between both algorithms on a variety of models, along with a comparison between the average time it took both algorithms to complete. I am aware that this method may also not be the best way to evaluate algorithm complexity and only presents a rough estimation. I will need to consult a specialist on that matter and conduct more tests but that would probably go beyond the scope of this article and should be contained in a separate paper focused only on the comparison between both methods.

 3. I have provided additional literature references to publications of the authors involved in the development of the structural number method where they descibe mentioned models in english. I hope that will provide the necessary background which was lacking previously.

 4. I agree and i can already attest that the method is currently being verified empirically. The synthesis method has been verified already with three real piezoelectric stacks with different geometrical dimensions which has shown some discrepancy between model results and real plate behaviour. The source of those differences was traced back to how some of the piezoelectric parameters are determined in the factory (under 1kHz wave stimulation) versus plate properties in the resonance proximity. However, i would like to envelope the empirical analysis, along with all the necessary test procedures and the test stand description in a second article, serving as the continuation for this study.

 I hope that i have provide an exhaustive explanation to the issues raised.

 Best Regards,
